# Solubility Data, Solubility Parameters and Thermodynamic Behavior of an Antiviral Drug Emtricitabine in Different Pure Solvents: Molecular Understanding of Solubility and Dissolution

**DOI:** 10.3390/molecules26030746

**Published:** 2021-01-31

**Authors:** Faiyaz Shakeel, Nazrul Haq, Ibrahim Alsarra, Sultan Alshehri

**Affiliations:** Department of Pharmaceutics, College of Pharmacy, King Saud University, P.O. Box 2457, Riyadh 11451, Saudi Arabia; faiyazs@fastmail.fm (F.S.); nazrulhaq59@gmail.com (N.H.); ialsarra@ksu.edu.sa (I.A.)

**Keywords:** dissolution, emtricitabine, molecular understanding, solubility, solubility parameter, thermodynamics

## Abstract

The solubility values, various Hansen solubility parameters (HSPs) and thermodynamic behavior of emtricitabine (ECT) in twelve different pure solvents (PS) were estimated using various experimental as well as computational methods. Experimental solubility values (*x*_e_) of ECT in twelve different PS were obtained at *T* = 298.2 K to 318.2 K and *p* = 0.1 MPa. The *x*_e_ values of ECT were correlated by “van’t Hoff, Apelblat and Buchowski-Ksiazaczak *λh* models”. Various HSPs for ECT and twelve different PS were also calculated using “HSPiP software”. The *x*_e_ values of ECT were estimated maximum in polyethylene glycol-400 (PEG-400; 1.41 × 10^−1^), followed by ethylene glycol, Transcutol-HP, propylene glycol, methanol, water, isopropanol, ethanol, 1-butanol, dimethyl sulfoxide, 2-butanol and EA (1.28 × 10^−3^) at *T* = 318.2 K. “Apparent thermodynamic analysis” showed an “endothermic and entropy-driven dissolution” of ECT. Overall, PEG-400 was found as the best/ideal solvent for solubility/miscibility of ECT compared to other solvents studied.

## 1. Introduction

**Figure 1:**
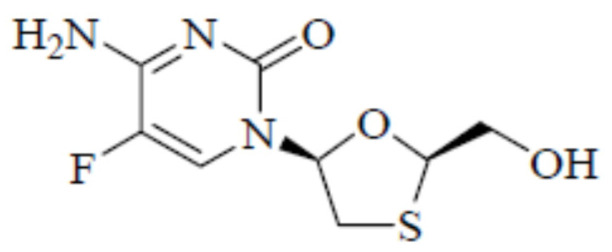
Molecular structure of emtricitabine (ECT) (molar mass: 247.24 g mol^−1^).

Emtricitabine (ECT, Figure 1; chemical name: 4-amino-5-fluoro-1-[(2*R*,5*S*)-2-(hydroxymethyl)-1,3-oxathiolan-5-yl]pyrimidin-2-one; molecular formula: C_8_H_10_FN_3_O_3_S; molar mass: 247.24 g mol^−1^, CASRN: 143491-57-0 and PubChem CID: 60877) occurs as a white to off white powder [1,2].

It is a synthetic nucleoside analog which is active against human immunodeficiency virus type I (HIV-I) reverse transcriptase [2,3]. It is used alone or in combination with other antiviral drugs in the treatment of HIV-I-infected patients [3,4,5]. ECT is commercially available as capsules or oral solutions by the brand name of Emtriva^®^ for the treatment of HIV-I-infected patients [2,5]. It is reported as freely soluble in water at *T* = 298.2 K [2]. ECT is highly water-soluble drug and hence rapidly and extensively absorbed following oral administration of capsules or oral solutions [5]. The absolute bioavailability of Emtriva^®^ capsules and Emtriva^®^ solution have been reported as 93% and 75%, respectively [2]. The relative bioavailability of Emtriva^®^ oral solutions has been reported as approximately 80% compared with capsules [2]. Various dosage form strategies such as solid dosage form [4,6,7,8], semi-solid gels [9], vesicle-based drug delivery system [10], microparticulate-based drug delivery system [11] and nanotechnology-based drug delivery systems [5,12,13,14,15] have been investigated in literature to improve its antiviral therapy. The quantitative solubility value of ECT in water has been reported as 112 mg mL^−1^ at *T* = 298.2 K [2]. Recently, the solubility values of ECT in mole fraction in neat water, neat polyethylene glycol-400 (PEG-400) and various (PEG-400 + water) mixtures at *T* = 298.2 K to 318.2 K and *p* = 0.1 MPa have been reported in literature [16]. While, temperature-dependent solubilities and other physicochemical parameters of ECT in other studied solvents are not reported elsewhere. The solubility and other physicochemical data of drugs in pure solvents have significant role in drug discovery process and formulation development [17,18,19]. Although, ECT had no solubility issues but the solubility and other physicochemical data of ECT are scarce in literature. ECT is a BCS class I drug but solubility data are important for any kind of drug in order to obtain the physicochemical information about those drugs [4,17]. The solubility data of drugs are important for various industrial processes such as purification, recrystallization, drug development, drug discovery, pre-formulation studies and formulation development [18,19]. Because the solubility data of ECT in most of the studied solvents is not reported in literature, this drug was chosen for this study. Therefore, the solubility values of ECT in various pure solvents (PS) namely “water, methanol, ethanol, isopropanol (IPA), 1-butanol, 2-butanol, ethylene glycol (EG), propylene glycol (PG), PEG-400, Transcutol-HP (THP), ethyl acetate (EA) and dimethyl sulfoxide (DMSO)” at *T* = 298.2 K to 318.2 K and *p* = 0.1 MPa were obtained using a saturation shake flask method. The solubility studies were carried out at different temperature under atmospheric pressure. The influence of pressure on ECT solubility was not performed in this study. The value of atmospheric pressure was not measure/maintained as the influence of pressure on solubility was not studied. The control of atmospheric pressure is not important in such cases. Normal and three-dimensional Hansen solubility parameters were calculated to select the best solvents for ECT. “Apparent thermodynamic analysis” was conducted to study dissolution behavior of ECT. The molecular interactions between ECT and various PS were also evaluated. The solubility values, solubility parameters and other physicochemical data of ECT obtained in this study would be useful in “purification, recrystallization, drug discovery, pre-formulation studies and formulation development” of ECT.

## 2. Results and Discussion

### 2.1. Solid Phase Characterization of ECT

**Figure 2:**
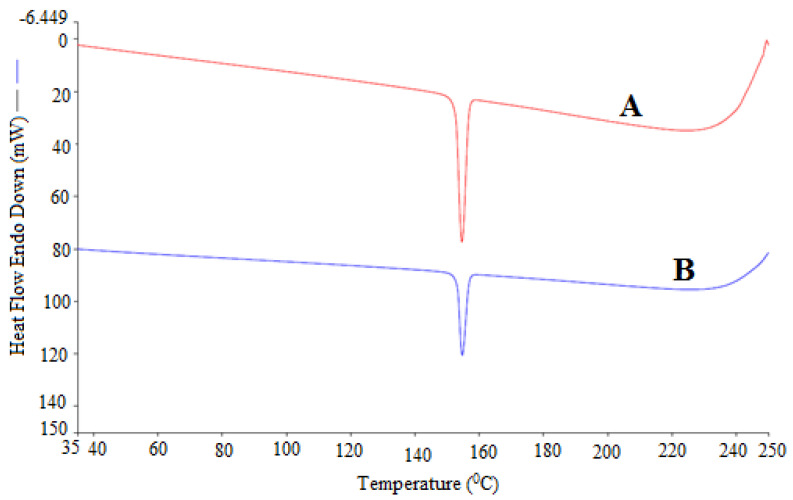
DSC spectra of (A) pure ECT and (B) equilibrated ECT (recovered from ethanol).

The solid phase characterization of ECT in pure and equilibrated sample was conducted using a differential scanning calorimetry (DSC) analysis. The representative DSC curves of pure and equilibrated ECT are given in Figure 2. The DSC curve of pure ECT showed a sharp crystalline peak at fusion temperature (*T*_fus_) = 154.60 °C (converted to 427.80 K). The fusion enthalpy (∆*H*_fus_) and heat capacity difference (∆*C*_p_) values of pure ECT were recorded as 32.37 kJ mol^−1^ and 75.66 J mol^−1^ K^−1^, respectively. The DSC curve of equilibrated ECT also showed a sharp crystalline peak at *T*_fus_ = 154.75 °C (converted to 427.95 K). The ∆*H*_fus_ and ∆*C*_p_ values of equilibrated ECT were recorded as 31.75 kJ mol^−1^ and 74.19 J mol^−1^ K^−1^, respectively. The DSC curves and various thermal parameters (i.e., *T*_fus_, ∆*H*_fus_ and ∆*C*_p_) of pure and equilibrated ECT were not varied significantly (*p* > 0.05), suggesting that ECT exists in pure crystalline form which was not transformed into polymorphs/hydrates/solvates after solubility experiments [18,20]. In addition, the X-ray diffraction (XRD) patterns of pure and equilibrated ECT were also found to be similar in our previous publication [16]. Hence, the DSC spectra of present study and XRD patterns of our previous publication suggested no transformation of ECT into different forms. Because the physical form of ECT remained unchanged from the sample recovered from ethanol, it was expected that its physical form will also be remain unchanged if the sample will be recovered from some other PS such as methanol, IPA, PEG-400, THP, water etc. The *T*_fus_ of ECT was recorded as 156.90 °C (converted to 430.10 K) by Srilatha et al. [6]. Fathima et al. found the *T*_fus_ of ECT as 153.40 °C (converted to 426.60 K) [11]. The *T*_fus_ of pure ECT was obtained as 154.60 °C (converted to 427.80 K) in the present work which was very close with those reported in literature [6,11].

### 2.2. Solubility Data of ECT Using Experimental Approach and Comparison with Literature

The experimental mole fraction solubility (*x*_e_) values of ECT in twelve different PS at *T* = 298.2 K to 318.2 K and *p* = 0.1 MPa were obtained by applying Equation (1) and results are listed in Table 1.

**Figure 3:**
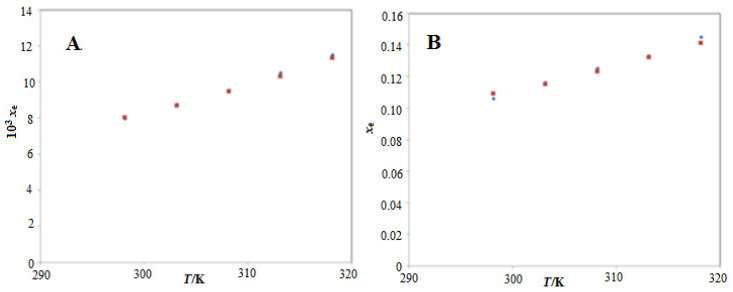
Comparison of mole fraction solubility values of ECT in (**A**) water and (**B**) PEG-400 with literature values at *T* = 298.2 K to 318.2 K; the symbol 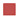 indicates the experimental mole fraction solubilities of ECT in (**A**) water and (**B**) PEG-400 and the symbol 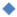 indicates the literature solubilities of ECT in (**A**) water and (**B**) PEG-400 taken from reference [16].

The solubility of ECT in water at *T* = 298.2 K is reported elsewhere [2]. The mole fraction solubility values of ECT in water, PEG-400 and various (PEG-400 + water) mixtures at *T* = 298.2 K to 318.2 K and *p* = 0.1 MPa have also been reported in our previous article [16]. However, the quantitative solubility values of ECT in other investigated PS are not reported elsewhere. The solubility of ECT in water at *T* = 298.2 K has been reported as 112 mg/mL (converted to 8.09 × 10^−3^ in mole fraction) [2]. The temperature dependent solubility data of ECT in any of the investigated PS are not reported by other researchers. Hence, we compared the temperature dependent experimental solubility data of ECT in neat water and neat PEG-400 with those reported in our previous publication. The mole fraction solubility values of ECT in water at *T* = 298.2 K to 318.2 K and *p* = 0.1 MPa have been reported in the range of 7.95 × 10^−3^ to 1.15 × 10^−2^ in our previous publication [16]. The mole fraction solubility of ECT in water at *T* = 298.2 K was calculated as 8.02 × 10^−3^ in the present work. The mole fraction solubility values of ECT in water at *T* = 298.2 K to 318.2 K and *p* = 0.1 MPa were calculated in the range of 8.02 × 10^−3^ to 1.13 x 10^−2^ in the present work. The mole fraction solubility values of ECT in PEG-400 at *T* = 298.2 K to 318.2 K and *p* = 0.1 MPa have been reported in the range of 1.06 × 10^−1^ to 1.45 × 10^−1^ in our previous publication [16]. The mole fraction solubility values of ECT in PEG-400 at *T* = 298.2 K to 318.2 K and *p* = 0.1 MPa were calculated in the range of 1.09 × 10^−1^ to 1.41 × 10^−1^ in the present work. The graphical correlation between experimental and literature solubility values of ECT in water (Figure 3A) and PEG-400 (Figure 3B) at *T* = 298.2 K to 318.2 K is summarized in Figure 3. The results summarized in Figure 3 suggested good correlation of experimental solubility values of ECT with its literature values. Overall, the solubility values of ECT in water and PEG-400 recorded in this work were found to be very close with those reported in literature [2,16].

The solubility values listed in Table 1 showed that the *x*_e_ values of ECT were found to be increasing significantly with the raise in temperature in all PS evaluated (*p* < 0.05). The *x*_e_ values of ECT were found highest in PEG-400 (1.41 × 10^−1^), followed by EG (5.72 × 10^−2^), THP (4.65 × 10^−2^), PG (3.90 × 10^−2^), methanol (1.49 × 10^−2^), water (1.13 × 10^−2^), IPA (8.19 × 10^−3^), ethanol (7.76 × 10^−3^), 1-butanol (6.84 × 10^−3^), DMSO (6.59 × 10^−3^), 2-butanol (6.55 × 10^−3^) and EA (1.28 × 10^−3^) at *T* = 318.2 K. In the present research, the impact of PEGs molar mass/polymerization on ECT solubility was not investigated because only PEG-400 was investigated in this research. In addition, PEG-400 is the most commonly used solubilizer among other grades of PEGs [16,21,22]. Nevertheless, the mole fraction solubility of drugs is known to be increased with increase in the molar mass of the solvents/co-solutes. Accordingly, in the present research, the mole fraction solubility of ECT would be enhanced with increase in the molar mass of PEGs [23]. Due to extensive use of PEG-400 in solubility enhancement, this grade was selected in this research [21,22]. Overall, the solubility of ECT was found good in many PS including PEG-400, EG, THP, PG, methanol and water.

The solubility values of solute also depend on the dielectric constant (K_d_) of PS. Hence, the influence of K_d_ on ECT solubility at *T* = 298.2 K was also studied in this work. The K_d_ values of different PS have been found in literature [24,25,26]. The K_d_ value of ECT is not reported in literature. However, it has been reported as freely soluble in water [2]. The literature K_d_ values for different PS at T = 298.2 K are summarized in Table 2. Among methanol, ethanol and IPA, the solubility of ECT was found to be maximum in methanol which might be due to medium K_d_ value of methanol compared to ethanol and IPA. The solubility of ECT was observed in similar magnitude in 1-butanol and 2-butanol which might be due to slightly difference in their K_d_ values. Between EG and PG, the solubility of ECT was slightly higher in EG which might be due to higher K_d_ value of EG compared to PG.

The K_d_ value of EA is the lowest compared to other PS but ECT solubility was also recorded lowest in EA, which might be due to the fact that ECT is not a nonpolar drug. Although, the K_d_ value of water was highest among other PS studied, ECT solubility was not recorded as highest or lowest in water. This observation suggested that the solubility of solutes not depend only on polarity/dielectric constant but also on molar mass, H-bonding and inter and intramolecular interactions between the solute and solvents. According to Equation (1) mentioned in the experimental section, the mole fraction solubility of solute is directly proportional to the molar mass of the PS. Hence, the maximum solubility of ECT in mole fraction in PEG-400 was possible due to its higher molar mass and lower dielectric constant compared to other PS studied.

### 2.3. Estimation of Solubility Parameters Using Computational Approaches

The values of various Hansen solubility parameters (HSPs) of ECT and different PS were obtained by applying Equation (2) and results are presented in Table 3.

The HSPs were obtained by the HSPiP software. The value of total HSP (*δ*) for ECT was predicted as 25.90 MPa^1/2^, showing that ECT had medium polarity that means it had both hydrophilic as well as lipophilic properties. Generally, the *x*_e_ values of ECT were found higher in PS with lower or medium HSP namely PEG-400, EG, THP, PG and methanol (Table 3). The polar HSP (*δ*_p_) of solutes, their dipole moment (*µ*) and molecular volume are associated with polarity. The values of molecular volume and *µ* of the aqueous solution of ECT have been found as 2.30 Å^3^ and 9.10 D, respectively in literature [27]. The *δ*_p_ value for ECT was found to be 12.90 MPa^1/2^ in this research. Overall, the literature values of molecular volume, *µ* and predicted value of ECT in this research indicated that ECT had medium polarity.

The value of the Van Krevelen and Hoftyzer solubility parameter (∆$\bar{\delta }$) was obtained using Equation (3). The value of ∆$\bar{\delta }$ < 5.0 was suggested for higher solubility of solute with solvent [28,29]. Many PS including DMSO (∆$\bar{\delta }$ = 5.90 MPa^1/2^), THP (∆$\bar{\delta }$ = 6.10 MPa^1/2^), ethanol (∆$\bar{\delta }$ = 6.73 MPa^1/2^), IPA (∆$\bar{\delta }$ = 7.32 MPa^1/2^), 1-butanol (∆$\bar{\delta }$ = 7.32 MPa^1/2^), PEG-400 (∆$\bar{\delta }$ = 7.42 MPa^1/2^) and 2-butanol (∆$\bar{\delta }$ = 7.89 MPa^1/2^) were found to very close with the range of ∆ $\bar{\delta }$ < 5.0 MPa^1/2^, suggesting ECT had good solubility in these solvents according to this concept. The value of three-dimensional (3D) solubility parameter space (*R*_a_) was calculated using Equation (4). The value of *R*_a_ < 5.6 has been recommended for higher solubility of solute with solvent [20,30]. Some PS including DMSO (*R*_a_ = 6.06 MPa^1/2^), THP (*R*_a_ = 6.93 MPa^1/2^), ethanol (*R*_a_ = 7.57 MPa^1/2^), 1-butanol (*R*_a_ = 8.34 MPa^1/2^), IPA (*R*_a_ = 8.42 MPa^1/2^) and 2-butanol (*R*_a_ = 8.92 MPa^1/2^) were found to very close with the range of *R*_a_ < 5.6 MPa^1/2^, suggesting ECT had good solubility in these solvents according to this concept. Another solubility parameter, the Greenhalgh’s solubility parameter (∆*δ*) was obtained using Equation (5). The value of ∆*δ* < 7.0 MPa^1/2^ has been recommended for higher solubility of solute with solvent [31]. Most of the PS such as ethanol (∆*δ* = 0.50 MPa^1/2^), DMSO (∆*δ* = 2.30 MPa^1/2^), 1-butanol (∆*δ* = 3.00 MPa^1/2^), 2-butanol (∆*δ* = 3.10 MPa^1/2^), PG (∆*δ* = 3.30 MPa^1/2^), IPA (∆*δ* = 3.60 MPa^1/2^), methanol (∆*δ* = 4.40 MPa^1/2^), THP (∆*δ* = 4.50 MPa^1/2^), EG (∆*δ* = 5.70 MPa^1/2^), PEG-400 (∆*δ* = 7.00 MPa^1/2^) and EA (∆*δ* = 7.80 MPa^1/2^) were found to very close with the range of ∆*δ* < 7.0 MPa^1/2^, suggesting ECT had good solubility in these solvents according to this concept. Overall, different solubility trend of ECT was observed based on various solubility parameter approaches. The highest solubility of ECT was recorded in PEG-400 and lowest solubility was observed in EA. Although, the different HSPs of PEG-400 and EA were not found lowest and highest, there is a certain relationship between the solubility and HSPs. The solubility of drugs depends not only on HSPs parameters but also depends on other parameters namely molar mass of the solute and solvent, intermolecular interactions between the solute and solvents and intramolecular interactions between the solute and solute and solvents and solvents, etc. [20]. The highest solubility of ECT in PEG-400 could be possible due to higher molar mass, higher H-bonding interactions between ECT and PEG-400 and lower dielectric constant. Overall, PEG-400 was found as the best solvent for solubility/miscibility of ECT.

### 2.4. Ideal Solubilities and Activity Coefficients

The “activity coefficients (*γ*_i_)” were estimated to study the molecular interactions between ECT and respective PS. The “ideal solubility (*x*^idl^)” values for ECT were obtained by applying Equation (6) and results are presented in Table 1. The *x*^idl^ values of ECT were calculated as 3.74 × 10^−2^ to 6.76 × 10^−2^ at *T* = 298.2 K to 318.2 K. The *x*^idl^ values of ECT were found very closed with *x*_e_ values of ECT in EG at each temperature studied (*p* > 0.05). However, the *x*^idl^ values of ECT were found significantly lower than *x*_e_ values of ECT in PEG-400 (*p* < 0.05). On the other hand, the *x*^idl^ values of ECT were observed slightly higher than *x*_e_ values of ECT in THP, PG and methanol. While, the *x*^idl^ values of ECT were found significantly higher than *x*_e_ values of ECT in EA, 1-butanol, 2-butanol, DMSO, ethanol, IPA and water (*p* < 0.05). Based on these results, PEG-400 was chosen as the ideal solvent for solubility/miscibility of ECT.

The values of *γ*_i_ for ECT in twelve different PS at *T* = 298.2 K to 318.2 K were calculated by applying Equation (8) and results are presented in Table 4.

The values of *γ*_i_ for ECT were calculated < 1.0 in PS such as PEG-400 and EG at each temperature studied. The values of *γ*_i_ for ECT were found much closed to 1.0 in PG and THP at each temperature level. While, the *γ*_i_ value for ECT was observed significantly higher in EA. The influence of K_d_ on *γ*_i_ values of ECT was also evaluated. The K_d_ values of various PS at *T* = 298.2 K are included in Table 2. Among methanol, ethanol and IPA, the *γ*_i_ value of ECT was found to be lowest in methanol. The *γ*_i_ value of ECT was observed slightly lower in 1-butanol compared to 2-butanol which was possible due to slightly difference in their K_d_ values. Between EG and PG, the *γ*_i_ value of ECT was slightly lower in EG which might be due to higher K_d_ value of EG compared to PG. Overall, the results of activity coefficients were found in same trend of solubility data and dielectric constants. Based on the data of activity coefficients, the highest molecular interactions were seen in ECT-PEG-400, ECT-EG, ECT-THP and ECT-PG in comparison with other combination of ECT and PS.

### 2.5. Computational Approaches for Solubility Correlation

**Figure 4:**
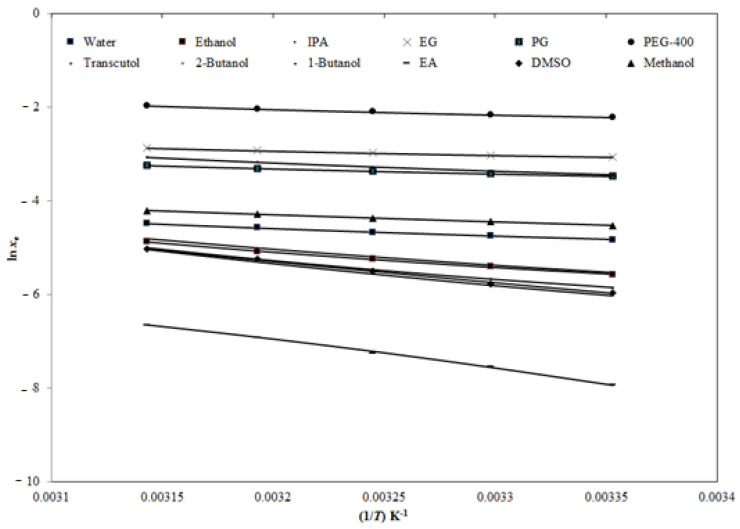
Correlation of experimental natural logarithmic solubilities (ln *x*_e_) of ECT with the Apelblat model in various pure solvents as a function of 1/*T*; symbols represent the experimental ln *x*_e_ values of ECT and the solid lines represent the ln *x*^Apl^ values calculated by the Apelblat model.

The *x*_e_ values of ECT recorded in this work were correlated with three different computational models such as the Apelblat, van’t Hoff and Buchowski-Ksiazaczak *λh* models [20,32,33,34,35]. The *x*_e_ and Apelblat solubility (*x*^Apl^) values of ECT were calculated using Equations (1) and (9), respectively. The graphical correlation of ln *x*_e_ values of ECT with its ln *x*^Apl^ values in twelve different PS as a function of 1/*T* is shown in Figure 4. The data summarized in Figure 4 suggested good correlation of ln *x*_e_ values of ECT with its ln *x*^Apl^ values in all twelve different PS.

The Apelblat correlation results are listed in Table 5.

Different Apelblat model parameters like *A, B* and *C* were determined from experimental solubility data of ECT. The values of *x*^Apl^ for ECT were back calculated using model parameters. The correlation between *x*_e_ and *x*^Apl^ values of ECT was performed using root mean square deviation (*RMSD*) and determination coefficient (*R*^2^) values. The *RMSD* value was calculated using Equation (10). The *RMSD* values for ECT in twelve different PS were obtained as (0.21 to 1.96)%. The overall *RMSD* value was calculated as 0.87%. The regressed *R*^2^ values for ECT in twelve different PS were calculated as 0.9977 to 0.9999. The lower values of *RMSD* and higher *R*^2^ values showed good correlation of *x*_e_ values of ECT with the Apelblat model.

The van’t Hoff solubility (*x*^van’t^) values of ECT were calculated using Equation (11). The van’t Hoff model parameters (*a* and *b*) were calculated from experimental solubility values of ECT. The *x*^van’t^ values of ECT were back calculated using model parameters. The graphical correlation of ln *x*_e_ values of ECT with its ln *x*^van’t^ values in twelve different PS as a function of 1/*T* is given in supplementary Figure S1 (Figure S1). The data summarized in Figure S1 also showed good correlation of ln *x*_e_ values of ECT with its ln *x*^van’t^ values. The results of van’t Hoff correlation are listed in Table 6. The *RMSD* values for ECT in twelve different PS were obtained as (0.67 to 2.39)%. The overall *RMSD* was calculated as 1.42%. The *R*^2^ values for ECT in twelve different PS were estimated as 0.9942 to 0.9987. The lower *RMSD* values and higher *R*^2^ values again suggested good correlation of *x*_e_ values of ECT with “van’t Hoff model”.

The Buchowski-Ksiazaczak *λh* model solubility (x) values of ECT were predicted using Equation (12) and results are summarized in Table 7. The *RMSD* values for ECT in twelve different PS were predicted as (0.79 to 2.84)%. The overall *RMSD* was predicted as 1.65%. The lower *RMSD* values again indicated good correlation of *x*_e_ values of ECT with Buchowski-Ksiazaczak *λh* model. Overall, the performance of each studied theoretical model was excellent for the correlation of experimental solubility data of ECT based on recorded *RMSD* values. However, the Apelblat model was found to be the best theoretical model for solubility correlation due to lowest *RMSD* value.

### 2.6. Thermodynamic Analysis

The obtained values of various apparent thermodynamic parameters of ECT along with *R*^2^ values are listed in Table 8. The values of apparent standard enthalpy (Δ_sol_*H*^0^) for ECT were calculated by applying Equation (13). The “Δ_sol_*H*^0^ values” for ECT dissolution in twelve different PS were calculated as (8.09 to 49.75) kJ mol^−1^. Generally, the Δ_sol_*H*^0^ values of ECT were found maximum in PS with lower solubilities of ECT such as EA, DMSO, 1-butanol and 2-butanol etc. However, the Δ_sol_*H*^0^ values of ECT were found minimum in PS with higher solubilities of ECT such as PEG-400, EG, THP and PG. The values of apparent standard Gibbs free energy (Δ_sol_*G*^0^) for ECT were calculated by applying Equation (14). The “Δ_sol_*G*^0^ values” for ECT dissolution were calculated as (5.34 to 18.57) kJ mol^−1^.

The Δ_sol_*G*^0^ value of ECT was found minimum in PEG-400 (5.34 kJ mol^−1^) which was possible due to the highest solubility of ECT in PEG-400. The Δ_sol_*G*^0^ value of ECT was found maximum in EA (18.57 kJ mol^−1^) which was possible due to the lowest solubility of ECT in EA. The results of Δ_sol_*G*^0^ value of ECT were found in accordance with solubility trend of ECT in twelve different PS. The recorded data of Δ_sol_*H*^0^ and Δ_sol_*G*^0^ for ECT in twelve different PS suggested an endothermic dissolution of ECT in all these PS [36,37]. The values of apparent standard entropy (Δ_sol_*S*^0^) for ECT were estimated using Equation (15). The Δ_sol_*S*^0^ values for ECT dissolution were calculated as (1.03 to 101.20) J mol^−1^ K^−1^, suggesting an entropy-driven dissolution of ECT in all PS studied [37]. The mean relative uncertainties in Δ_sol_*H*^0^, Δ_sol_*G*^0^ and Δ_sol_*S*^0^ for ECT dissolution were calculated as 0.59, 0.31 and 0.90, respectively. Overall, the dissolution of ECT has been considered as an endothermic and entropy-driven in all twelve PS investigated [36,37].

## 3. Materials and Methods

### 3.1. Materials

ECT, methanol, ethanol, IPA, 1-butanol and 2-butanol were obtained from Sigma Aldrich (St. Louis, MO, USA). THP was donated as a kind gift sample from Gattefosse (Lyon, France). EG, PG, PEG-400, EA and DMSO were obtained from Fluka Chemica (Busch, Switzerland). Water was collected from a Milli-Q unit. All the materials (including ECT) provided by the supplier were of high purity and used without any further purification. The information and properties of materials are listed in Table S1.

### 3.2. Analysis of ECT

The analysis of ECT in all in vitro samples was conducted using a reversed phase high performance liquid chromatography (HPLC) system connected with an ultraviolet (UV) detector at *λ*_max_ = 254 nm. The chromatographic quantification of ECT was carried out at *T* = 298.2 K using a Waters HPLC system (Waters, Milford, MA, USA). A Nucleodur (150 × 4.6 mm, 5 μm) RP C_18_ column was used for reversed phase HPLC analysis of ECT. The binary mixture of ethanol and methanol (50:50%) was utilized as the mobile phase. The mobile phase was flowed with a flow rate of 1.0 mL min^−1^ at 254 nm for the elution of ECT. The volume of injection volume and retention time was 20 µL and 1.6 min, respectively. The calibration curve (CC) of ECT was obtained by plotting the concentrations of ECT against recorded HPLC area. The CC of ECT was found linear in the range of (0.1–100) µg g^−1^ with *R*^2^ value of 0.9992. The regression equation for ECT was recorded as *y* = 18,911*x* + 12,028; in which *x* and y represent the concentration of ECT and measured HPLC area, respectively. This regression equation was applied for the estimation of ECT concentration in unknown solubility samples.

### 3.3. Solid Phase Characterization of ECT

The solid phase characterization of ECT in its pure form and equilibrated sample was performed by DSC. The thermal parameters, crystallinity and any transformation of ECT were evaluated using DSC technique. Equilibrated ECT was obtained from equilibrated sample of ethanol containing ECT by its slow evaporation [17,18]. DSC investigation of pure and equilibrated ECT was performed using a DSC-8000 instrument (Perkin Elmer, Akron, OH, USA). The whole assembly was equipped a with chiller (*T* = 253.2 K) and autosampler. For DSC analysis, accurately weighed 4.50 mg of pure and equilibrated ECT were taken into aluminum pan which were sealed hermetically. The flow rate of nitrogen was set at 20 mL min^−1^ for the analysis of both samples. The samples were heated with 10 K min^−1^ heating rate in the range of *T* = 303.2 to 523.2 K for both samples.

### 3.4. Solubility Measurement of ECT

The solubilities of ECT in twelve different PS were measured using an experimental approach by applying a saturation shake flask technique at *T* = 298.2 K to 318.2 K and *p* = 0.1 MPa [38,39]. The operation and experimental procedure of this study were same as reported in previously published studies [17,20]. Hence, the experimental conditions are not described here. Each experiment was carried out in triplicates manner (*n* = 3.0) in order to establish good statistical analysis. The concentration of ECT in experimental saturated samples of each PS was estimated by HPLC technique at *λ*_max_ = 254 nm. The amount of ECT (μg g^−1^) was obtained from the CC of ECT discussed under Section 3.2. Finally, the *x*_e_ values of ECT were obtained using the following equation [19,20]:

(1) $$\;\;\;\;\;x_{e}=\;\frac{m_{1}/M_{1}}{m_{1}/M_{1}+m_{2}/M_{2}}$$

Here, *m*_1_ = mass of ECT; *m*_2_ = mass of PS; *M*_1_ = molar mass of ECT and *M*_2_ = molar mass of PS.

### 3.5. Estimation of Solubility Parameters Using Computational Approaches

The solute at a standard condition of temperature and pressure reaches highest solubility/miscibility in PS having the similar solubility parameter [40]. Hence, different solubility parameters for ECT and twelve different PS were calculated in this work. The value of total HSP was calculated by applying following equation [20,40,41]:

(2) $$\delta^{2}=\delta_{d}^{2}+\;\delta_{p}^{2}+\;\delta_{h}^{2}$$

Here, *δ* = total HSP; *δ*_d_ = dispersion HSP; *δ*_p_ = polar HSP and *δ*_h_ = hydrogen-bonded HSP. The values of different HSPs for ECT and twelve different PS were estimated using HSPiP software (version 4.1.07, Louisville, KY, USA).

However, the ∆$\bar{\delta }$ value was calculated by applying the following equation [28]:

(3) $$∆\bar{\delta }=\;{\left[\left(\delta_{d}{}_{2}^{2}-\delta_{d}{}_{1}^{2}\right)+\left(\delta_{p}{}_{2}^{2}-\delta_{p}{}_{1}^{2}\right)+\left(\delta_{h}{}_{2}^{2}-\delta_{h}{}_{1}^{2}\right)\right]}^{1/2}$$

It has been reported that if ∆$\bar{\delta }$ < 5.0 MPa^1/2^, the possibility of miscibility between solute and solvent is maximum [28,29].

The value of *R*_a_ was obtained by applying the following equation [30,42]:

(4) $$Ra^{2}=4{\left(\delta_{d2}-\delta_{d1}\right)}^{2}+\;{\left(\delta_{p2}-\delta_{p1}\right)}^{2}+{\left(\delta_{h2}-\delta_{h1}\right)}^{2}$$

Here, subscript 1 and 2 refer to the respective PS and ECT, respectively. For maximum miscibility between the solute and PS, the *R*_a_ value should be <5.6 MPa^1/2^ [30].

The ∆*δ* value was obtained by applying the following equation [31]:

(5) $$∆\delta =\;\delta_{2}-\delta_{1}$$

It was suggested that the miscibility between solute and PS is higher at ∆*δ* < 7.0 MPa^1/2^. While, the value of ∆*δ* > 10.0 MPa^1/2^ is suggested for insolubility between the solute and solvent [20,31].

### 3.6. Ideal Solubilities and Activity Coefficients

The *x*^idl^ value of ECT was calculated by applying the following equation [43]:

(6) $$\ln\;x^{\mathrm{idl}}=\;\frac{-∆H_{\mathrm{fus}}\left(T_{\mathrm{fus}}-T\right)}{RT_{\mathrm{fus}}T}+\left(\frac{∆C_{p}}{R}\right)[\frac{T_{\mathrm{fus}}-T}{T}+\ln\left(\frac{T}{T_{\mathrm{fus}}}\right)]\;$$

Here, *R* = universal gas constant and other parameters have already been explained in previous text. The ∆*C*_p_ for ECT was calculated by applying the following equation [43,44]:

(7) $$∆C_{p}=\;\frac{∆H_{\mathrm{fus}}}{T_{\mathrm{fus}}}$$

The *T*_fus_ and ∆*H*_fus_ values for ECT were calculated as 427.80 K and 32.37 kJ mol^−1^, respectively using thermal (i.e., DSC) analysis. Using Equation (7), the ∆*C*_p_ of ECT was obtained as 75.66 J mol^−1^ K^−1^. The *x*^idl^ values for ECT were now obtained by applying Equation (6).

The *γ*_i_ values for ECT in twelve different PS were estimated by applying the following equation [43,45]:

(8) $$\gamma_{i}=\;\frac{x^{\mathrm{idl}}}{x_{e}}$$

The γ_i_ data for the solute in various PS are used for the description of drug-solvent interactions at molecular level. If the values of γ_i_ are lower, the drug-solvent interactions at molecular level will be higher and vice verse.

### 3.7. Solubility Validation/Correlation Using Computational Approaches

Computational analysis of experimental solubility value of drugs is important for their practical predictions and validations [38,46]. Hence, the experimental solubility values of ECT in twelve different PS were correlated using three computational models namely the Apelblat, van’t Hoff and Buchowski-Ksiazaczak *λh* models [20,32,33,34,35]. According to the Apelblat model, the solubility of solute depends on the temperature and it can be used to correlate the experimental solubility data of both polar as well as non-polar solutes [32]. It represents the relationship between the solubility and temperature in semi-empirical form. The *x*^Apl^ value of ECT was estimated by applying the following equation [32,33]:

(9) $$\ln\;x^{\mathrm{Apl}}=A+\frac{B}{T}+\;C\ln\left(T\right)$$

Here, *A, B* and *C* = the parameters of the Apelblat model and the values of these parameters were estimated by applying nonlinear multivariate regression analysis of experimental solubilities of ECT listed in Table 1 [20]. These parameters reflect the influence of non-ideality of the solution on ECT mole fraction solubility. The *x*_e_ values of ECT were correlated with *x*^Apl^ values of ECT in terms of *RMSD* and *R*^2^ values. The *RMSD* for ECT was obtained by applying the following equation:

(10) $$\;\;\;\;\;\;\;\;\;\;\;\;\;\;\;\;\;\;\;\;\;RMSD=\;{\left[\frac{1}{N}\overset{N}{\underset{i=1}{\sum }}{\left(\frac{x^{\mathrm{Apl}}-x_{e}}{x_{e}}\right)}^{2}\right]}^{\frac{1}{2}}$$

Here, *N* = the number of data points used in the experiment.

The van’t Hoff model is used to describe the relationship between the solubility of solute with reciprocal of temperature and used to correlate the experimental solubility data of polar solutes. It is also a semi-empirical model. The *x*^van’t^ value of ECT was obtained by applying the following equation [20]:

(11) $$\ln\;x^{\mathrm{van}’t}=a+\frac{b}{T}$$

Here, *a* and *b* = the parameters of the van’t Hoff model and the values of these parameters were estimated by plotting ln *x*_e_ values of ECT against 1/*T*.

The Buchowski-Ksiazaczak *λh* model is the other model used to describe solution behavior and correlation of solubility data. This model can be used to fit the experimental data well for various systems with only two parameters i.e., *λ* and *h* [34]. The Buchowski-Ksiazaczak *λh* model solubility of ECT was obtained using the following equation [34,35]:

(12) $$\ln\;[1+\frac{\lambda \left(1-x\right)}{x}]\;=\;\lambda h\;[\frac{1}{T}-\frac{1}{T_{\mathrm{fus}}}]\;$$

Here, *λ* and *h* = the parameters of Buchowski-Ksiazaczak *λh* model and *x* = mole fraction solubility of ECT for Buchowski-Ksiazaczak *λh* model. The parameter *λ* represents the non-ideality of the solution.

### 3.8. Thermodynamic Analysis

For the calculation of thermodynamics parameters of ECT in twelve different PS, an apparent thermodynamic analysis was performed. Three thermodynamic parameters namely Δ_sol_*H*^0^, Δ_sol_*G*^0^ and Δ_sol_*S*^0^ for ECT were estimated by applying the van’t Hoff and Krug et al. analysis [43,47,48]. The Δ_sol_*H*^0^ values for ECT dissolution in twelve different PS were obtained at a mean harmonic temperature (*T*_hm_) of 308 K using “van’t Hoff analysis” by applying the following equation [43,47]:

(13) (∂ln xe∂(1T−1Thm))P= −∆solH0R

The Δ_sol_*H*^0^ values were estimated by “van’t Hoff” plots made between ln *x*_e_ values of ECT against 1T−1Thm. The representative van’t Hoff graphs are given in Figure S2.

The Δ_sol_*G*^0^ values for ECT dissolution in twelve different PS were also calculated at *T*_hm_ of 308 K using “Krug et al. analysis” by applying the following equation [47]:

(14) $${∆}_{\mathrm{sol}}G^{0}=\;-RT_{\mathrm{hm}}\times \mathrm{intercept}$$

Here, the intercept values for ECT in twelve different PS were calculated using van’t Hoff plots shown in Figure S2.

Finally, the Δ_sol_*S*^0^ values for ECT dissolution were obtained by applying the following equation [43,47,48]:

(15) $${∆}_{\mathrm{sol}}S^{0}=\;\frac{{∆}_{\mathrm{sol}}H^{0}-{∆}_{\mathrm{sol}}G^{0}}{T_{\mathrm{hm}}}$$

### 3.9. Statistical Analysis

Statistical evaluation was performed using Kruskal-Wallis analysis followed by Denn’s test. The “GraphpadInstat software (San Diego, CA, USA) was used to perform all statistical tests. The *p* < 0.05 was taken as significant value.

## 4. Conclusions

The solubility data, various solubility parameters and thermodynamic behavior of ECT in twelve different PS were obtained using different experimental and computational approaches. Experimental solubility values of ECT were measured using a saturation shake flask method. The solid phases of ECT in pure and equilibrated samples were characterized using DSC analysis. The results of DSC analysis showed no transformation of ECT in solvates/polymorphs/hydrates. The experimental solubilities of ECT were correlated well by the van’t Hoff, Apelblat and Buchowski-Ksiazaczak *λh* models with overall *RMSD* values of 1.42, 0.87 and 1.65%, respectively. Overall, the performance of each studied model was excellent. However, the Apelblat model was found to be the best theoretical model due to lowest *RMSD* value. The solubility of ECT was found to be increasing with the raise in temperature in all PS evaluated. The solubility of ECT was found maximum in PEG-400, followed by EG, THP, PG, methanol, water, IPA, ethanol, 1-butanol, DMSO, 2-butanol and EA at *T* = 318.2 K. The results of activity coefficients indicated higher molecular interactions in ECT-PEG-400, ECT-EG, ECT-THP and ECT-PG compared with other combination of ECT with PS. The data of apparent thermodynamic analysis indicated an endothermic and entropy-driven dissolution of ECT in all PS studied. Overall, PEG-400 was found as the best solvent for solubility/miscibility of ECT compared to other solvents investigated.

## Figures and Tables

**Table 1:** Experimental solubilities (*x*_e_) of emtricitabine (ECT) in mole fraction in various pure solvents (*PS*) at *T* = 298.2 K to 318.2 K and *p* = 0.1 MPa ^a^.

*PS*	*x*_*e*_				
	*T* = 298.2 K	*T* = 303.2 K	*T* = 308.2 K	*T* = 313.2 K	*T* = 318.2 K
EA	3.63 × 10^−4^	5.34 × 10^−4^	7.12 × 10^−4^	9.96 × 10^−4^	1.28 × 10^−3^
2-Butanol	2.43 × 10^−3^	2.98 × 10^−3^	3.94 × 10^−3^	5.07 × 10^−3^	6.55 × 10^−3^
DMSO	2.56 × 10^-3^	3.15 × 10^−3^	4.09 × 10^−3^	5.34 × 10^−3^	6.59 × 10^−3^
1-Butanol	2.89 × 10^−3^	3.46 × 10^−3^	4.32 × 10^−3^	5.36 × 10^−3^	6.84 × 10^−3^
Ethanol	3.82 × 10^−3^	4.54 × 10^−3^	5.31 × 10^−3^	6.29 × 10^−3^	7.76 × 10^−3^
IPA	3.95 × 10^−3^	4.64 × 10^−3^	5.58 × 10^−3^	6.76 × 10^−3^	8.19 × 10^−3^
Water	8.02 × 10^−3^	8.66 × 10^−3^	9.45 × 10^−3^	1.03 × 10^−2^	1.13 × 10^−2^
Methanol	1.08 × 10^−2^	1.17 × 10^−2^	1.26 × 10^−2^	1.38 × 10^−2^	1.49 × 10^−2^
PG	3.18 × 10^−2^	3.27 × 10^−2^	3.47 × 10^−2^	3.67 × 10^−2^	3.90 × 10^−2^
THP	3.20 × 10^−2^	3.45 × 10^−2^	3.81 × 10^−2^	4.10 × 10^−2^	4.65 × 10^−2^
EG	4.66 × 10^−2^	4.87 × 10^−2^	5.12 × 10^−2^	5.39 × 10^−2^	5.72 × 10^−2^
PEG-400	1.09 × 10^−1^	1.15 × 10^−1^	1.23 × 10^−1^	1.32 × 10^−1^	1.41 × 10^−1^
x^idl^	3.74 × 10^−2^	4.35 × 10^−2^	5.05 × 10^−2^	5.85 × 10^−2^	6.76 × 10^−2^

^a^ The relative uncertainties *u*_r_ are *u*_r_(*T*) = 0.013, *u*_r_(*p*) = 0.003 and *u*_r_(*x*_e_) = 0.014.

**Table 2:** The literature values of dielectric constants (K_d_) for different PS at *T* = 298.2 K.

PS	K_d_	Reference
EA	6.00	[24]
2-Butanol	16.60	[25]
DMSO	47.00	[24]
1-Butanol	17.64	[25]
Ethanol	25.00	[24]
IPA	17.90	[24]
Water	79.00	[24]
Methanol	33.10	[26]
PG	32.00	[24]
THP	12.60	[24]
EG	37.00	[24]
PEG-400	14.10	[24]

**Table 3:** Various solubility parameters of ECT and various *PS* at *T* = 298.2 K.

Components	Hansen Solubility Parameters				*R*_a_*/MPa^1/2^	∆ $\bar{\delta }$ /MPa^1/2^	∆δ*/MPa^1/2^
	*δ*_d_/MPa^1/2^	*δ*_p_/MPa^1/2^	*δ*_h_/MPa^1/2^	*δ*/MPa^1/2^			
ECT	18.20	12.90	13.00	25.90	-	-	-
Water	15.50	16.00	42.30	47.80	29.95	29.58	21.90
EG	18.00	11.10	23.40	31.60	10.56	10.55	5.70
PG	17.40	9.10	21.70	29.20	9.62	9.52	3.30
Ethanol	16.20	8.40	17.60	25.40	7.57	6.73	0.50
IPA	15.80	6.60	14.30	22.30	8.42	7.32	3.60
1-Butanol	15.90	6.30	15.20	22.90	8.34	7.32	3.00
2-Butanol	15.80	5.40	12.40	20.80	8.92	7.89	3.10
PEG-400	14.60	7.50	9.40	18.90	9.69	7.42	7.00
THP	16.30	7.20	11.90	21.40	6.93	6.10	4.50
EA	15.70	5.60	7.00	18.10	10.69	9.77	7.80
Methanol	17.40	10.60	22.40	30.30	9.80	9.71	4.40
DMSO	17.40	14.20	7.30	23.60	6.06	5.90	2.30

* These values were calculated between ECT and respective *PS*.

**Table 4:** Activity coefficients (*γ*_i_) of ECT in various *PS* at *T* = 298.2 K to 318.2 K.

*PS*	*γ*_*i*_				
	*T* = 298.2 K	*T* = 303.2 K	*T* = 308.2 K	*T* = 313.2 K	*T* = 318.2 K
EA	103.18	81.56	71.00	58.73	52.78
2-Butanol	15.40	14.58	12.83	11.54	10.32
DMSO	14.58	13.83	12.36	10.95	10.25
1-Butanol	12.95	12.57	11.68	10.90	9.87
Ethanol	9.80	9.59	9.50	9.30	8.71
IPA	9.46	9.38	9.05	8.66	8.25
Water	4.66	5.02	5.34	5.64	5.94
Methanol	3.44	3.69	3.99	4.24	4.52
PG	1.20	1.33	1.45	1.59	1.73
THP	1.16	1.26	1.32	1.39	1.45
EG	0.80	0.89	0.98	1.08	1.18
PEG-400	0.34	0.37	0.40	0.44	0.47

**Table 5:** Results of the Apelblat model in terms of the Apelblat parameters (*A, B* and *C*), *R*^2^ and root mean square deviation (*RMSD*) for ECT in various *PS* ^b^.

*PS*	*A*	*B*	*C*	*R* ^2^	*RMSD* (%)	Overall *RMSD* (%)
EA	756.93	−40,177.60	−110.59	0.9993	1.96	
2-Butanol	−565.49	21,632.90	85.45	0.9991	1.42	
DMSO	−269.00	8184.21	41.34	0.9977	1.62	
1-Butanol	−704.53	28,585.44	105.80	0.9999	1.16	
Ethanol	−509.45	20,319.03	76.47	0.9989	1.15	
IPA	−464.21	18,103.22	69.84	0.9998	0.66	0.87
Water	−232.41	9030.47	34.62	0.9998	0.46	
Methanol	−95.77	2908.17	14.30	0.9994	0.24	
PG	−136.47	5194.27	20.28	0.9997	0.21	
THP	−303.07	12,231.01	45.38	0.9995	0.62	
EG	−193.44	7911.56	28.75	0.9999	0.51	
PEG-400	−191.99	7671.17	28.79	0.9998	0.54	

^b^ The relative uncertainties *u*_r_ are *u*_r_(*A*) = 0.60, *u*_r_(*B*) = 0.72 and *u*_r_(*C*) = 0.60.

**Table 6:** Results of van’t Hoff model in terms of model parameters (*a* and *b*), *R*^2^ and *RMSD* for ECT in various *PS* ^c^.

*PS*	*a*	*b*	*R* ^2^	*RMSD* (%)	Overall *RMSD* (%)
EA	12.14	−5976.00	0.9972	2.39	
2-Butanol	9.92	−4762.30	0.9970	1.98	
DMSO	9.36	−4578.30	0.9971	1.97	
1-Butanol	7.87	−4099.30	0.9955	2.08	
Ethanol	5.50	−3305.90	0.9953	1.81	
IPA	6.09	−3471.60	0.9971	1.40	1.42
Water	0.75	−1665.80	0.9969	0.71	
Methanol	0.52	−1507.20	0.9987	0.98	
PG	0.11	−1071.50	0.9974	1.06	
THP	2.55	−1790.60	0.9952	0.93	
EG	0.18	−972.49	0.9942	1.07	
PEG-400	1.88	−1223.40	0.9962	0.67	

^c^ The relative uncertainties *u*_r_ are *u*_r_(*a*) = 0.90 and *u*_r_(*b*) = 0.59.

**Table 7:** Results of the Buchowski-Ksiazaczak *λh* model in terms of model parameters (*λ* and *h*) and *RMSD* for ECT in various *PS* ^d^.

*PS*	*λ*	*h*	*RMSD* (%)	Overall *RMSD* (%)
EA	0.82	7255.94	2.84	
2-Butanol	0.20	22884.67	2.65	
DMSO	0.33	13682.90	2.24	
1-Butanol	0.70	5794.06	1.95	
Ethanol	1.22	2708.86	1.86	
IPA	1.02	3392.22	1.70	1.65
Water	2.14	777.68	1.58	
Methanol	1.99	755.86	1.28	
PG	1.38	773.75	1.15	
THP	0.63	2823.84	0.98	
EG	1.08	897.04	0.86	
PEG-400	0.02	50767.60	0.79	

^d^ The relative uncertainties *u*_r_ are *u*_r_(*λ*) = 0.68 and *u*_r_(h) = 1.55.

**Table 8:** Apparent thermodynamic parameters (Δ_sol_*H*^0^, Δ_sol_*G*^0^ and Δ_sol_*S*^0^) along with *R*^2^ values for ECT in various *PS* ^e^.

*PS*	Δ_sol_*H*^0^/kJ mol^−1^	Δ_sol_*G*^0^/kJ mol^−1^	Δ_sol_*S*^0^/J mol^−1^ K^−1^	*R* ^2^
EA	49.75	18.57	101.20	0.9971
2-Butanol	39.65	14.17	82.70	0.9971
DMSO	38.11	14.07	78.06	0.9972
1-Butanol	34.13	13.91	65.64	0.9957
Ethanol	27.52	13.37	45.92	0.9954
IPA	28.90	13.25	50.78	0.9972
Water	13.86	11.92	6.32	0.9970
Methanol	12.54	11.17	4.45	0.9988
PG	8.92	8.60	1.03	0.9975
THP	14.90	8.35	21.28	0.9954
EG	8.09	7.60	1.61	0.9944
PEG-400	10.18	5.34	15.71	0.9963

^e^ The relative uncertainties are *u*(Δ_sol_*H*^0^) = 0.59, *u*(Δ_sol_*G*^0^) = 0.31 and *u*(Δ_sol_*S*^0^) = 0.90.

## Data Availability

All the data associated with present manuscript have been included in supplementary materials.

## Supplementary Materials

The following are available online, Figure S1: Correlation of experimental natural logarithmic solubilities (ln *x*_e_) of emtricitabine (ECT) with the van’t Hoff model in various pure solvents (*PS*) as a function of 1/*T*; symbols represent the experimental ln *x*_e_ values of ECT and the solid lines represent the ln *x*^van’t^ values calculated by the van’t Hoff model, Figure S2: van’t Hoff plots for ECT plotted between ln *x*_e_ and 1/*T*−1/*T*_hm_ for ECT in various *PS*, Table S1: List of materials and their properties.

## Sample Availability

Samples of the compounds ECT are available from the authors.
