# Supplementary material for: Solubility Data, Solubility Parameters and Thermodynamic Behavior of an Antiviral Drug Emtricitabine in Different Pure Solvents: Molecular Understanding of Solubility and Dissolution
Source: Molecules. 2021 Jan 31;26(3):746. doi: 10.3390/molecules26030746 (PMC7867001; doi:10.3390/molecules26030746)
Supplement: Supplementary file 1 [file molecules-26-00746-s001.pdf]

Supporting information

Solubility Data, Solubility Parameters and Thermodynamic Behavior of an Antiviral Drug Emtricitabine in Different Pure Solvents: Molecular Understanding of Solubility and Dissolution

Faiyaz Shakeel, Nazrul Haq, Ibrahim A. Alsarra and Sultan Alshehri*

Department of Pharmaceutics, College of Pharmacy, King Saud University, P.O. Box: 2457, Riyadh 11451, Saudi Arabia; faiyazs@fastmail.fm (F.S.), nazrulhaq59@gmail.com (N.H.), ialsarra@ksu.edu.sa (I.A.)

* Correspondence: salshehri1@ksu.edu.sa (S.A.)

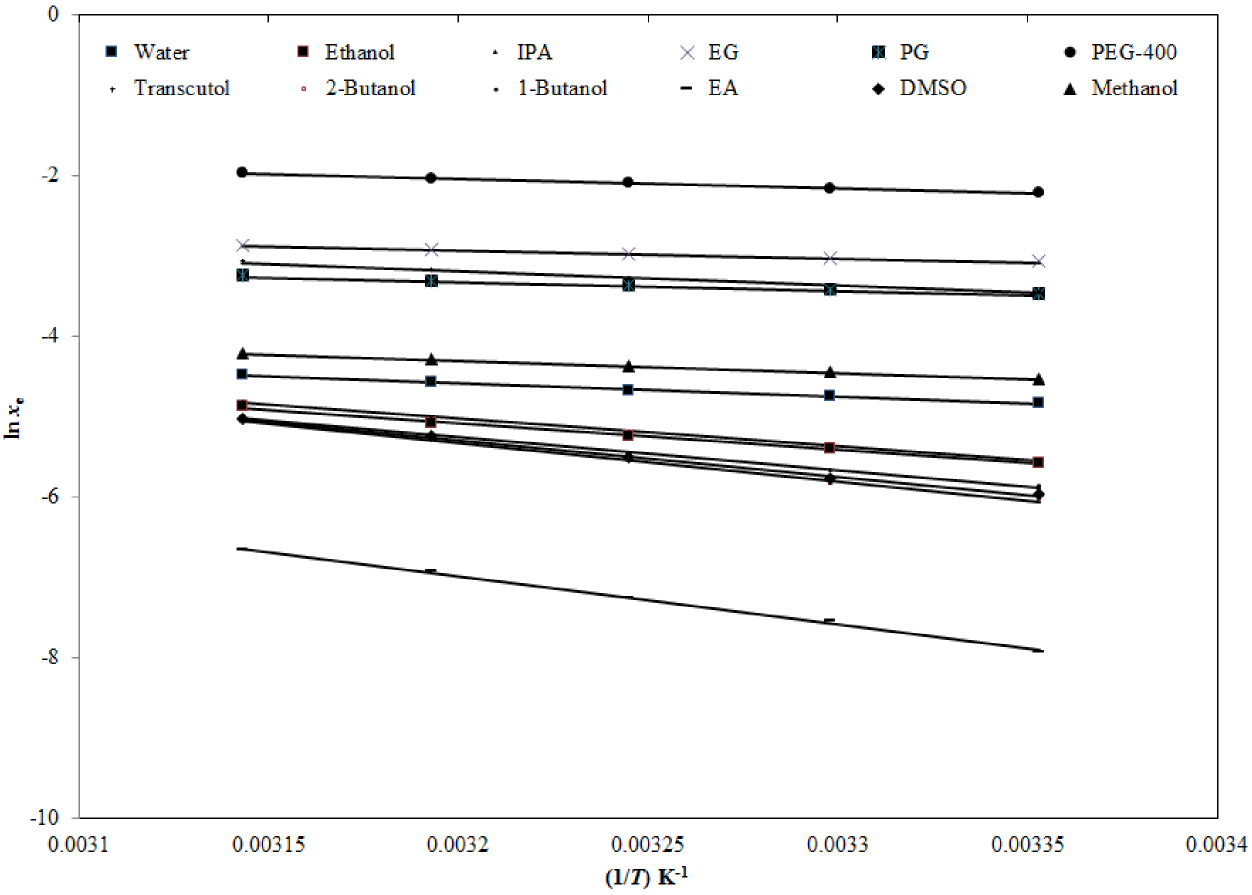

Figure S1. Correlation of experimental natural logarithmic solubilities (ln x_e) of emtricitabine (ECT) with "van't Hoff model" in various pure solvents (*PS*) as a function of 1/*T*; symbols represent the experimental ln x_e values of ECT and the solid lines represent the ln $x^{van't}$ values calculated by "van't Hoff model".

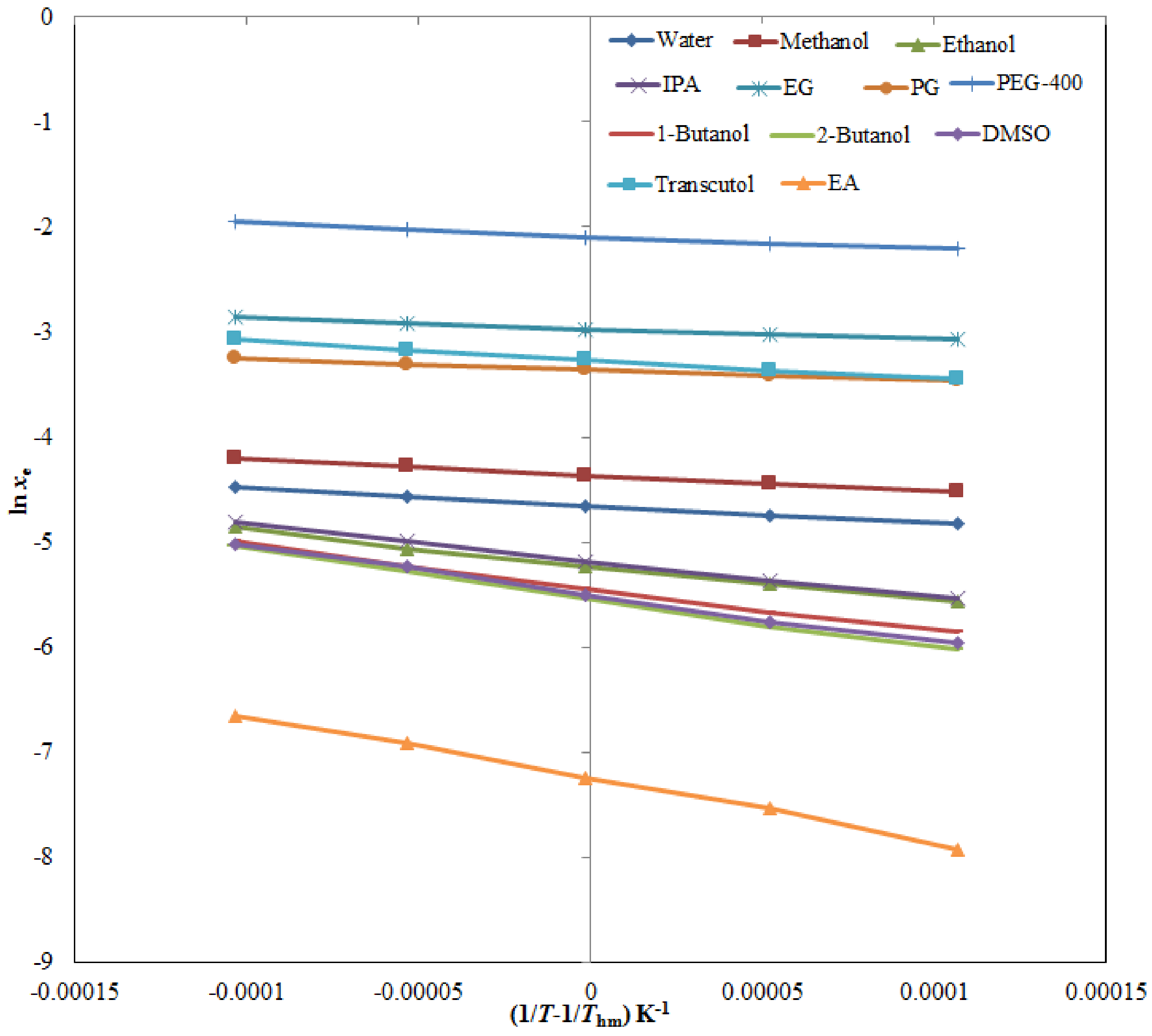

Figure S2. van't Hoff plots for ECT plotted between ln x_e and $1/T-1/T_{hm}$ for ECT in various *PS*.

Table S1: List of materials and their properties.

Material	Molecular formula	Molar mass (g mol^{-1})	CAS Registry no.	Purification method	Mass fraction purity	Analysis method	Source
ECT	$C_8H_{10}FN_3O_3S$	247.24	143491-57-0	None	>0.98	HPLC	Sigma Aldrich
Methanol	CH_3OH	32.04	67-56-1	None	>0.99	GC	Sigma Aldrich
Ethanol	C_2H_5OH	46.07	64-17-5	None	>0.99	GC	Sigma Aldrich
EG	$C_2H_6O_2$	62.07	107-21-1	None	>0.99	GC	Fluka Chemica
Transcutol	$C_6H_{14}O_3$	134.17	111-90-0	None	>0.99	GC	Gattefosse
PG	$C_3H_8O_2$	76.09	57-55-6	None	>0.99	GC	Fluka Chemica
PEG-400	$H(OCH_2CH_2)_nOH$	400	25322-68-3	None	>0.99	HPLC	Fluka Chemica
IPA	C_3H_8O	60.10	67-63-0	None	>0.99	GC	Sigma Aldrich
1-Butanol	$C_4H_{10}O$	74.12	71-36-3	None	>0.99	GC	Sigma Aldrich
2-Butanol	$C_4H_{10}O$	74.12	78-92-2	None	>0.99	GC	Sigma Aldrich
DMSO	C_2H_6OS	78.13	67-68-5	None	>0.99	GC	Fluka Chemica
EA	$C_4H_8O_2$	88.11	141-78-6	None	>0.99	GC	Fluka Chemica
Water	H_2O	18.07	7732-18-5	None	-	-	Milli-Q

Both the analysis method and purity were provided by supplier of each material.
